# Supplementary material for: Treatments with Liquid Smoke and Certain Chemical Constituents Prevalent in Smoke Reduce Phloem Vascular Sectoriality in the Sunflower with Improvement to Growth
Source: Int J Mol Sci. 2022 Oct 18;23(20):12468. doi: 10.3390/ijms232012468 (PMC9604246; doi:10.3390/ijms232012468)
Supplement: Supplementary file 1 [file ijms-23-12468-s001.zip › ijms-1943563-supplementary.pdf]

International Journal of
Molecular Sciences

MDPI

Supplemental Materials

Treatments with Liquid Smoke and Certain Chemical Constituents Prevalent in Smoke Reduce Phloem Vascular Sectoriality in the Sunflower with Improvement to Growth

Randi Noel, Mary Benoit, Stacy L. Wilder, Spenser Waller, Michael Schueller, and Richard A. Ferrieri

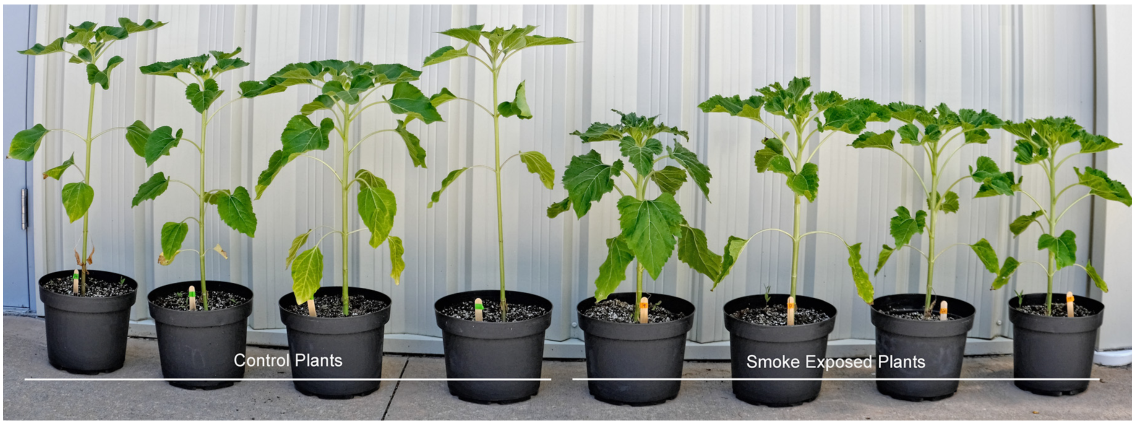

Figure S1: Photo of outdoor potted plants showing untreated control (left side) and liquid smoke-treated plants (right side).

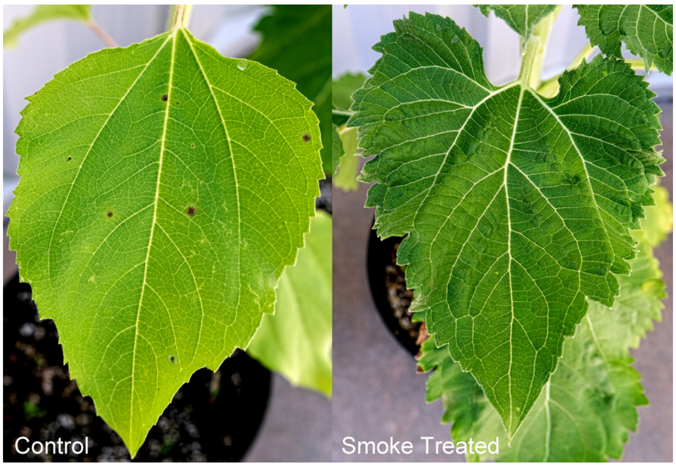

Figure S2: Closeup photo of a mature leaf from a control plant (left-side) and a mature leaf from a liquid smoke-treated plant (right-side) which shows a noticeable crinkled-leaf phenotype.

International Journal of
Molecular Sciences

MDPI

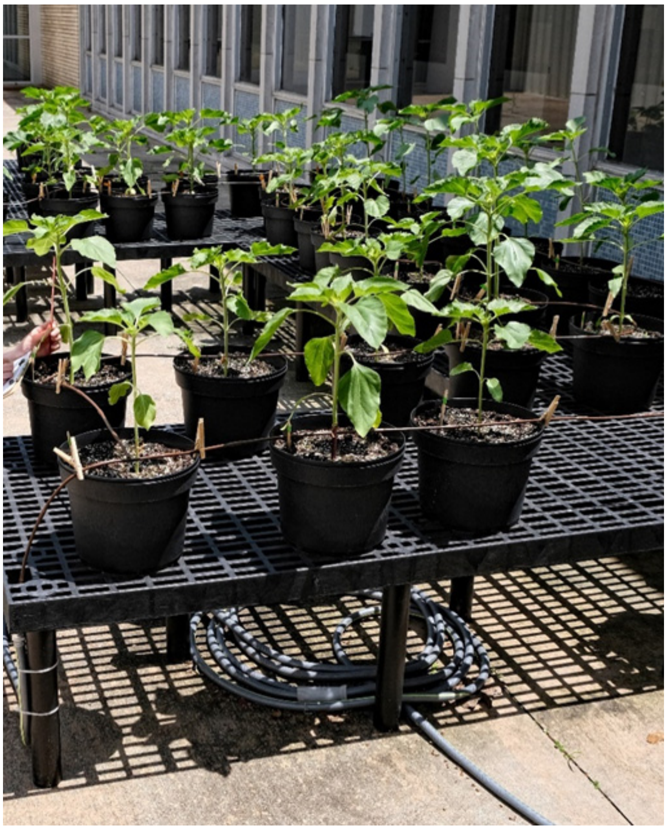

Figure S3: Photo showing potted plants on raised tables with drip irrigation tied into each pot.

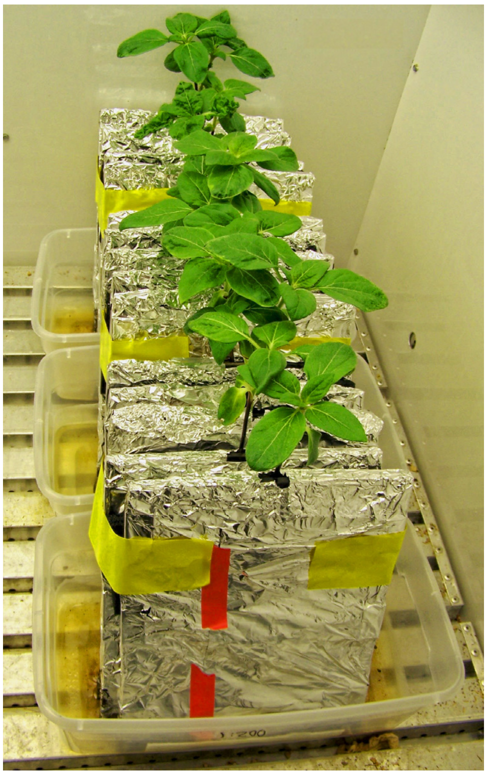

Figure S4: Sunflowers growing in rhizoboxes.
